# Clinical Outcomes of *In Vitro* Fertilization among Chinese Infertile Couples Treated for Syphilis Infection

**DOI:** 10.1371/journal.pone.0133726

**Published:** 2015-07-24

**Authors:** Jing Wang, Xiaomiao Zhao, Ping Yuan, Tingfeng Fang, Nengyong Ouyang, Ruiqi Li, Songbang Ou, Wenjun Wang

**Affiliations:** Reproductive Medicine Center, Department of Obstetrics & Gynecology, Sun Yat-Sen Memorial Hospital, Sun Yat-Sen University, No. 107 Yanjiang Xi Road, Guangzhou 510120, P. R. China; Center for Human Reproduction, UNITED STATES

## Abstract

To compare the clinical outcomes of infertile patients with and without syphilis after *in vitro* fertilization and embryo transfer (IVF-ET), in this case-control study, 320 infertile couples were enrolled and divided into syphilis (n = 160) and control groups (n = 160). The primary IVF outcomes were the clinical pregnancy rate and the birth of a healthy baby. All syphilis patients received the standard anti-syphilis treatment before undergoing IVF/ICSI. Our results showed that the endometrial thickness of the syphilis group was greater than that of the control group following hCG injection (16.9±5.4 vs. 13.0±4.7 mm, *P*<0.001). The numbers of normally fertilized eggs and normally cleaved fertilized eggs and the implantation rate were 6.8±4.8, 6.3±4.7 and 24.2%, respectively, for the syphilis group and 8.3±4.6, 8.1±4.6 and 34.4%, respectively, for the control group, and these values were significantly different between the groups. The clinical pregnancy rate was lower in the syphilis group compared with that in the control group (43.8% vs. 55.6%, *P* = 0.03). Lower offspring birth weight was observed in the infected male group compared with those in the infected female (2.7±0.4 vs. 3.0±0.4 kg, *P* = 0.01) and infected couple groups (2.7±0.4 vs. 3.1±0.5 kg, *P* = 0.007). Therefore, syphilis infection reduces the clinical pregnancy rate after IVF/ICSI.

## Introduction

Syphilis is a sexually transmitted disease (STD) caused by *Treponema pallidum*, and it is primarily spread through sexual intercourse[1,2], blood transfusion[3] or maternal-neonatal transmission[4]. The incidence rate of syphilis has risen rapidly in China since 1978[5,6], and it has become the most common STD in economically developed regions[7]. Syphilis infection of the reproductive tract[8]can cause inflammatory disease and infertility[9]. Reproductive tract infection and inflammation cause 8% and 35% of all cases of male infertility, respectively[10]. In women, it can result in fallopian tube obstruction[11,12] and endometritis. Approximately 72.4 million people worldwide[13] have infertility problems. In vitro fertilization (IVF) may be the last resort for couples attempting to overcome infertility[14,15]. The relationship between syphilis and infertility has been rarely reported in the literature. One previous report[16] on the effects of syphilis on IVF outcome has shown lower fertilization and implantation rates due to infection of the male parent with latent syphilis compared with the rates in those without syphilis infection. However, women with syphilis were not included in the study. Therefore, we performed retrospective analysis of patients who received IVF/intra-cytoplasmic sperm injection (ICSI) to evaluate the effects of syphilis on IVF-embryo transfer (ET) outcome.

## Materials and Methods

### Design

All patients in this study provided written informed consent for the use of their medical records. The Ethics Committee of the Sun Yat-Sen Memorial Hospital approved this study.The ethics committee approval of the Sun Yat-sen Memorial Hospital [2014] Record of ethics committee No. (02) Reproductive medicine centre: After censored by the medical ethics committee of the Sun Yat-sen Memorial Hospital, the clinical research project of your department “The effect of syphilis infection to outcomes of IVF: a retrospective case-control study” was approved to be carried out based on the retrospective study according with the requirements of medical ethics. Medical Ethics Committee 16 January 2014. In this retrospective case-control study, clinical data were obtained from 320 infertile couples presenting to Sun Yat-Sen Memorial Hospital between January 2008 and March 2014. The patients were divided into a syphilis group(n = 160) and a control group(n = 160)(age-matched at a ratio of 1:1). The syphilis group was divided into the following three subgroups: the infected male group (couples in which the male was anti-syphilis antibody-positive and the female was healthy, n = 69), the infected female group (couples in which the female was anti-syphilis antibody-positive and the male was healthy, n = 65), and the infected couple group (both partners were infected, n = 26). No interventional treatments were performed.

### Patient selection

Couples who failed to conceive after at least 1 year of intercourse without contraception were considered to be infertile. Infertility was caused by fallopian tube dysfunction and/or male-related factors (a diagnosis of asthenozoospermia and/or asthenoteratozoospermia or azoospermia according to the WHO Laboratory Manual for the Examination and Processing of Human Semen[17,18]). All patients underwent fresh ET during the first cycle and did not receive any medication for 3 months prior to IVF/ICSI. Patients were excluded if they had hysteromyoma, adenomyosis, ovarian tumor, endometriosis, polycystic ovarian syndrome (PCOS), hyperprolactinemia, thyroid disease, adrenal disease, diabetes, or a chromosomal aberration(determined by karyotype). Women with a reproductive tract abnormality or uterine malformation and those with a sperm or egg donor were also excluded.

Ovarian function was examined, and peripheral blood samples were collected on days 2–4 of menstruation to examine the basic follicle-stimulating hormone (bFSH), basic luteinizing hormone (bLH), and estrogen (E_2_) levels. Ultrasonography was performed to determine the antral follicle count (AFC). Routine semen and sperm morphology tests were conducted[15]. All participants received laboratory serologic examinations (the rapid plasma reagin [RPR] and/or *Treponema pallidum* particle agglutination [TPPA] tests) to test for the presence of syphilis infection prior to the IVF/ICSI treatment.

### Syphilis test

All serum samples were first assessed using a non-*Treponema pallidum* antigen serologic (RPR) test to screen for syphilis, and then a *Treponema pallidum* antigen serologic (TPPA) test was conducted to confirm the syphilis-positive samples identified by the screening test[19,20,21]. For the RPR test, cardiolipin is used as an antigen to detect anti-cardiolipin antibodies in the serum[22]. All serum samples for the RPR test were progressively diluted (1:1 to 1:32) in normal saline for titer determination[23]. In the TPPA test, gelatin granules sensitized to *Treponema pallidum* bind to anti-*Treponema pallidum* antibodies in human serum. The combination of the RPR and TPPA tests is widely used[24] for the maximal detection of syphilis during all phases of the disease with adequate sensitivity and specificity.

The patients were divided into the syphilis and control groups based on the laboratory serologic results. The patients in the syphilis group with a history of syphilis treatment (according to medical records) but no history of re-exposure did not receive syphilis treatment in this study[20]. An anti-syphilis treatment (penicillin G^20^) was administered to the patients without a history of syphilis infection and with positive RPR or TPPA results[20]. A 4-fold (2 dilutions) or greater decrease in the anti-*Treponema pallidum* antibody titer was indicative of treatment efficacy. This titer remained stable over a long period of time in the few patients who received the standard anti-syphilis treatment, a phenomenon known as “fixed serology”, if the patients without clinical signs of relapse were not treated with anti-syphilis therapy. The patients received IVF/ICSI treatment at one month after the disappearance of their clinical syphilis symptoms or after their *Treponema pallidum* antigen serologic test results became negative. Some patients remained positive for *Treponema pallidum*-specific antibody after the anti-syphilis treatment. Couples were included in the control group if both patients showed negative RPR and TPPA results.

### Controlled ovarian hyperstimulation

The mid-luteal long protocol was employed as follows [25]: a 1.87 mg dose of long-acting GnRH-a (Diphereline; Ipsen Pharma Biotech, Signes, France) was administered during the prior mid-luteal menstrual cycle to promote pituitary downregulation, as determined byblood E_2_ <50 ng/L, LH <5 IU/L, FSH <5 IU/L, and ultrasound-detected vaginal endometrial thickness <5 mm. Gonadotropin (Gn) was administered at 14 days after downregulation to promote follicle development. The Gn dosage was determined based on patient age, the basal FSH level, BMI, and the AFC, and 150–300 IU/d recombinant human FSH (Gonal-F; Merck Serono, Geneva, Switzerland) was administered. During ovarian hyperstimulation, the blood E_2_, progesterone, and LH levels were measured, and follicle development was monitored by transvaginal ultrasound (ALOKA 3500, Japan). When three follicles with ≥16 mm diameters, two follicles with ≥17 mm diameters, or one follicle with a ≥18 mm diameter was detected on ultrasound, 10,000 IU human chorionic gonadotropin (hCG) (Livzon, Zhuhai, China) was administered on that evening. Ultrasound-guided transvaginal oocyte retrieval was conducted at 34 to 36 hours after hCG injection, and a sperm sample was concurrently collected and subjected to density gradient centrifugation[15]. The fertilization regimen and traditional IVF and ICSI fertilization protocols used were selected based on the concentration and motility of the washed sperm. No special ovulation or sperm washing procedures were performed for the antibody-positive patients without a recent syphilis reinfection.

### Embryo grading and transfer

Embryo morphology evaluations were conducted by monitoring the pronuclei for 17±1 hours after fertilization. Embryo cleavage and development were monitored for 44±1 hours and 68±1 hours after fertilization, respectively[26]. Optimal embryos that met the quality screening criteria did not exhibit multiple pronuclei; rather, they showed 4–5 blastomeres of homogenous sizes with an accumulation of <5% cell debris by day 2 and 7–9 blastomeres of homogenous sizes with an accumulation of <5% cell debris by day 3. Ultrasound-guidedET was conducted on day 3 after oocyte retrieval. When available, two embryos were transferred into the women who were <35 years old, and three embryos were transferred into those who were ≥35 years old.

### Luteal support

Luteal support was achieved via daily intramuscular injections of progesterone (60 mg/day) (Progesterone Injection, Xian Ju Pharmaceutical Co., Zhejiang, China) following oocyte retrieval for up to 14 days after ET. Serum β-hCG was tested on day 14 after ET, and progesterone administration was continued for 12 weeks after diagnosis of pregnancy. Ultrasonography was conducted at 35 days after ET, and clinical pregnancy was diagnosed via the presence of an intrauterine gestational sac, fetal bud, and fetal heartbeat; otherwise, biochemical diagnosis was performed.

## Statistical Analyses

### Data collection

We reviewed the patients’ medical records to obtain information about their hospital and telephone follow-up assessments.

The collected data were analyzed using SPSS 21.0. A total of 3,275 couples with no history of syphilis infection were included. We selected 160 patients from the 3,275 couples to form the control group. First, the 3,275 couples were divided into 20 groups based on age (range of 24–43 years), according to the age range of the syphilis group, and then random patient numbers were generated for each age group using SPSS 21.0 with the seed set to (1,100) and inclusion of four decimal places in the random numbers. According to the number of patients in each syphilis age group, 160 patients were extracted [27] from the randomly generated numbers in ascending order by stratified random sampling to form the control group. The t-test was used to perform between-group comparisons of the normally distributed quantitative datasets with homogeneous variances, and the results are presented as the mean ± standard deviation. The Mann-Whitney test was conducted to analyze the non-normally distributed datasets, and the results are presented as medians with ranges. Analysis of variance was applied to compare the means of the three quantitative subgroups when that data for each sample was normally distributed with a homogeneous variance. The least significant difference (LSD) test was used to perform pairwise comparisons between any two of the three groups, and the results are presented as the mean ± standard deviation. The Kruskal-Wallis test was conducted to assess non-normally distributed data among the three subgroups or those that did not exhibit homogeneous variances, and the results are presented as medians with ranges. The chi-square test was carried out to assess the qualitative data, and the results are reported as percentiles. Binary analyses were conducted using logistic regression, and factors were introduced into the regression equation using the Enter method. All results were considered significant (two-tailed) at *P*<0.05.

## Results

### Clinical characteristics of the syphilis and control groups

No significant differences were found in female age (*P* = 0.99), duration of infertility (*P* = 0.50), BMI (*P* = 0.15), or the basal FSH (*P* = 0.12), basal LH (*P* = 0.59) or basal E_2_ level (*P* = 0.81) between the syphilis and control groups. Endometrial thickness on the hCG injection day was significantly higher for the syphilis group compared with the control group (16.9±5.4 vs. 13.0±4.7 mm, *P*<0.001). Syphilis infection likely affected endometrial thickness (Table 1).

**Table 1:** Demographic and cycle characteristics of patients diagnosed with syphilis and their respective healthy controls.

Characteristic	Syphilis group (n = 160)	Control group (n = 160)	*T* ^d^ */Z* ^e^	*P* value
Female age (y)^a^	32.4±4.0	32.4±4.0	0.01	0.99
Duration of infertility (y)^a^	5.0±3.6	5.3±3.7	-0.7	0.50
BMI (kg/m^2^)^a^	21.1±2.3	21.6±2.8	-1.5	0.15
Basal FSH (IU/L)^a^	8.4±3.5	7.9±2.4	1.6	0.12
Basal LH (IU/L)^a^	4.3±2.0	4.4±2.0	-0.5	0.59
Basal E_2_ (pg/mL)^a^	41.8±17.4	42.3±19.1	-0.2	0.81
E_2_ on HCG day (pg/mL)^a^	2611.1±1492.3	2745.2±1512.1	-0.8	0.43
Endometrial thickness on HCG day (mm)^a^ ^b^	16.9±5.4	13.0±4.7	6.9	<0.001
Total dose of administered gonadotropins (IU)^a^	2125.0±752.3	2201.8±809.5	-0.9	0.38

Note: BMI = body mass index; FSH = follicle-stimulating hormone; LH = luteinizing hormone.

a The independent samples t-test was conducted to compare the syphilis and control groups, and the values are presented as the mean ± standard deviation.

b A significant difference was detected between the syphilis and control groups using the independent samples t-test (*P*<0.05).

### Laboratory parameters and clinical outcomes of the syphilis and control groups

The number of normal fertilizations (6.8±4.8 vs. 8.3±4.6, *P* = 0.004), number of oocytes with normal cleavage (6.3±4.7 vs. 8.1±4.6, *P* = 0.001) and implantation rate (24.2% vs. 34.4%, *P* = 0.003) significantly differed between the syphilis and control groups. The clinical pregnancy rate was significantly lower in the syphilis group compared with the control group (43.8% vs. 55.6%, *P* = 0.03), and the biochemical pregnancy rate was higher in the syphilis group (7.5% vs. 0.6%, *P* = 0.002) (Table 2).

**Table 2:** Laboratory parameters and clinical outcomes of the syphilis and control groups.

Characteristic	Syphilis group (n = 160)	Control group (n = 160)	*T* ^d^ */Z* ^e^ /*X* ^*2*^	*P* value
Total number of retrieved follicles^a^	11.2±6.8	12.3±6.6	-1.4	0.17
Number of retrieved mature oocytes^a^	9.2±6.0	10.4±5.5	-1.9	0.06
Number of normally fertilized oocytes^a^ ^b^	6.8±4.8	8.3±4.6	-2.9	0.004
Number of fertilized oocytes with normal cleavage^a^ ^b^	6.3±4.7	8.1±4.6	-3.4	0.001
Normal fertility rate (%)^a^	86.8±17.3	85.0±15.1	1.0	0.31
Number of high-quality embryos^c^	1.0 (0–11)	1.0 (0–11)	-0.3	0.78
Number of embryos transferred^c^	2.0 (1–3)	2.0 (1–3)	-2.7	0.007
Implantation rate (%)^d^ ^e^	24.2 (80/330)	34.4 (123/357)	8.6	0.003
Live birth rate per embryos transferred (%)^d^	49.3 (75/152)	54.9 (107/195)	1.0	0.31
Clinical pregnancy rate (%)^d^ ^e^	43.8 (70/160)	55.6 (89/160)	4.5	0.03
Biochemical pregnancy rate (%)^d^ ^e^	7.5 (12/160)	0.6 (1/160)	9.7	0.002
Early miscarriage rate (%)^f^	5.7 (4/70)	4.5 (4/89)	─	0.73
Deformity rate (%)^f^	0.13 (1/75)	0 (0/107)	─	0.41
Fetal birth weight (kg)^a^	2.8±0.5	2.8±0.5	0.6	0.52
Average gestational period^a^	37.3±1.6	37.0±2.0	1.0	0.33
Fetal birth weight at delivery <37 weeks (kg)^a^	2.6±0.4	2.5±0.5	0.6	0.52
Fetal birth weight at delivery ≥37 weeks (kg)^a^	3.0±0.5	3.0±0.5	0.09	0.93
Average gestational period at delivery <37 weeks^a^	35.4±0.7	35.1±1.6	0.9	0.36
Average gestational period of delivery ≥37 weeks^a^	38.1±1.1	38.1±1.2	0.2	0.82

Note: The quality screening criteria for high-quality embryos included the presence of 7–8 blastomeres of equal size with an accumulation of <20% cell debris by day 3 (Embryology, 2011).

Normally fertilized oocytes were defined as those with two pronuclei (2PN) and 2 polar bodies at 16–20 hours after oocyte retrieval.

The normal fertilization rate was calculated as follows: number of normally fertilized oocytes / (number of oocytes with one pronucleus + number with two pronuclei + number with multiple pronuclei + number with late cleavage).

The implantation rate (IR) was defined as the number of gestational sacs per number of embryos transferred.

The live birth rate per embryos transferred was defined as the total number of births per the number of embryos transferred that resulted in clinical pregnancy.

The clinical pregnancy rate per embryo transferred was determined by the number of patients with a gestational sac in the uterus at 5 weeks after embryo transfer.

Early miscarriage was defined as the natural termination of pregnancy before 12 weeks, even in the presence of a positive serum hCG test or an ultrasound-detected intrauterine gestational sac after embryo transfer.

Biochemical pregnancy was defined as a pregnancy that did not clinically progress, accompanied by a β-hCG level of ≥25 U/L.

a The independent samples t-test was performed to compare the syphilis and control groups, and the values are presented as the mean ± standard deviation.

b A significant difference was detected between the syphilis and control groups using the independent samples t-test (*P*<0.05).

c The Mann-Whitney test was conducted to compare the non-normally distributed data, and the results are shown as the median (range).

d Data were obtained using Pearson’s chi-square test.

e A significant difference was detected between the syphilis and control groups using Pearson’s chi-square test (*P*<0.05).

^f^ Data were obtained using Fisher’ Exact Test.

### Clinical characteristics, laboratory parameters, and clinical outcomes of the three subgroups

No significant differences in age (*P* = 0.25), the duration of infertility (*P* = 0.23), basal FSH level (*P* = 0.52), basal E_2_ level (*P* = 0.31), total dosage of Gn (*P* = 0.79), number of retrieved oocytes (*P* = 0.38), or early miscarriage rate (*P* = 0.81) were found among the infected women, infected men, and infected couple subgroups. Fetal birth weight (*P* = 0.006) significantly differed among the three subgroups. Furthermore, the LSD test revealed that the fetal birth weight was significantly lower for the offspring of the infected males compared with those of the infected females (2.7±0.4 vs. 3.0±0.4 kg, *P* = 0.01) and couples (2.7±0.4 vs. 3.1±0.5 kg, *P* = 0.007). The average gestational period was also shorter in the infected male group compared with the infected female (36.5±1.4 vs. 37.9±1.5 kg, *P<*0.001) and couple groups (36.5±1.4 vs. 38.1±1.6 kg, *P* = 0.003). Fetal birth weight at delivery <37 weeks was significantly higher in the couple group compared the infected female (3.3±0.3 vs. 2.7±0.4 kg, *P* = 0.01) and infected male groups (3.3±0.3 vs. 2.4±0.2 kg, *P* = 0.001). No differences in the clinical pregnancy rate were detected among the subgroups (women: 43.1% vs. men: 44.9% vs. couples: 42.3%, *P* = 0.96) (Table 3).

**Table 3:** Clinical characteristics, laboratory parameters, and clinical outcomes of the three groups.

Characteristic	Infected female group (n = 65)	Infected male group (n = 69)	Infected couple group (n = 26)	*F/x* ^*2*^	*P* value
Female age (y)^a^	32.7±3.9	32.6±4.3	31.2±3.3	1.4	0.25
Duration of infertility (y)^a^	5.5±4.1	4.8±3.4	4.2±2.7	1.5	0.23
BMI (kg/m^2^)^a^ ^b^	20.6±2.0	21.6±2.5*	21.2±2.4	3.4	0.04
Basal FSH (IU/L^)^ ^c^	7.8 (1.3–28.4)	7.5 (4.1–16.9)	9.0 (5.0–17.0)	1.3	0.52
Basal E_2_ (pg/mL)^a^	41.4±18.1	43.7±17.6	37.6±14.9	1.2	0.31
Basal LH (IU/L)^c^	4.3 (1.2–14.6)	3.8 (1.2–8.0)	3.8 (1.9–8.0)	2.5	0.29
E_2_ on HCG day (pg/mL)^a^	2562.1±1406.2	2672.0±1611.4	2571.8±1421.2	0.1	0.91
Endometrial thickness on HCG day (mm)^a^	18.0±5.0	15.8±5.3	16.9±6.0	2.9	0.06
Total dose of administered gonadotropins (IU)^a^	2077.1±745.1	2166.3±801.1	2135.4±649.8	0.2	0.79
Total number of retrieved follicles^a^	10.4±6.9	11.7±6.6	12.3±7.0	1.0	0.38
Number of retrieved mature oocytes^a^	8.3±5.2	10.5±6.5	8.3±5.9	2.8	0.07
Number of normally fertilized oocytes^a^	6.1±4.0	7.6±5.1	6.3±5.5	1.6	0.20
Number of fertilized oocytes with normal cleavage^a^	5.7±4.0	7.1±5.0	5.6±5.6	1.8	0.18
Normal fertilization rate (%)^a^	89.8±15.4	84.9±18.4	84.5±18.6	1.6	0.21
Number of high-quality embryos^c^	1.0 (0–8)	1.0 (0–11)	1.0 (0–4)	0.4	0.82
Number of embryos transferred^c^	2.0 (1–3)	2.0 (1–3)	2.0 (1–3)	2.2	0.34
Implantation rate (%)^d^	22.6 (31/137)	25.9 (37/143)	24.0 (12/50)	0.4	0.82
Live birth rate per embryos transferred (%)^d^	49.2 (30/61)	51.5 (35/68)	43.5 (10/23)	0.4	0.80
Clinical pregnancy rate (%)^d^	43.1 (28/65)	44.9 (31/69)	42.3 (11/26)	0.07	0.96
Biochemical pregnancy rate (%)^f^	6.2 (4/65)	8.7 (6/69)	7.7 (2/26)	0.43	0.92
Early miscarriage rate (%)^f^	3.6 (1/28)	6.5 (2/31)	9.1 (1/11)	1.0	0.81
Fetal birth weight (kg)^a^ ^b^	3.0±0.4	2.7±0.4*	3.1±0.5^&^	5.6	0.006
Average gestational period^a^ ^b^	37.9±1.5	36.5±1.4*	38.1±1.6^&^	8.8	<0.001
Fetal birth weight at delivery <37 weeks (kg)^a^ ^b^	2.7±0.4	2.4±0.2	3.3±0.3^#^ ^&^	8.6	0.002
Fetal birth weight at delivery ≥37 weeks (kg)^a^	3.0±0.4	2.9±0.4	3.0±0.6	0.7	0.52
Average gestational period of delivery <37 weeks^a^	35.6±0.5	35.3±0.7	36.0±0.0	1.3	0.29
Average gestational period of delivery ≧37 weeks^a^	38.3±1.1	37.7±0.8	38.6±1.3	3.0	0.06

Note: Multiple groups of quantitative data were compared using analysis of variance, and the LSD test was performed for pairwise comparisons between any two of the three groups.

a The variance and LSD test were used to compare the three subgroups, and the values are presented as the mean ± standard deviation.

b A significant difference was detected among the three subgroups by analysis of variance (*P*<0.05).

c Non-normally distributed data are presented as the median (range), as assessed by the Kruskal-Wallis test.

d Data were obtained using Pearson’s chi-square test.

^f^ Data were obtained using Fisher’s Exact Test.

* A significant difference was detected between the infected female and infected male groups.

^#^ A significant difference was detected between the infected female and infected couple groups.

^&^A significant difference was detected between the infected male and infected couple groups.

### Logistic regression analysis of the clinical pregnancy rate

Logistic regression analysis was performed to evaluate the confounding factors for the clinical pregnancy rate. We found that the presence of syphilis (OR: 0.615, 95% CI: 0.382–0.991, *P* = 0.046), female age (OR: 0.895, 95% CI: 0.838–0.955, *P* = 0.001), and BMI (OR: 1.124, 95% CI: 1.023–1.235, *P* = 0.015) all influenced this rate (Table 4).

**Table 4:** Logistic regression analysis of clinical pregnancy.

	B	SE	Wald	df	Sig.	Exp(B)	95% CI for Exp(B)	
							Lower	Upper
Step 1^a^Presence or absence of syphilis	-0.486	0.243	3.991	1	0.046	0.615	0.382	0.991
Female age	-0.111	0.033	11.211	1	0.001	0.895	0.838	0.955
BMI	0.117	0.048	5.908	1	0.015	1.124	1.023	1.235
Duration of infertility	-0.004	0.034	0.013	1	0.909	0.996	0.932	1.065
Basal FSH	0.025	0.046	0.304	1	0.581	1.026	0.937	1.122
Basal LH	0.104	0.068	2.368	1	0.124	1.110	0.972	1.267
Basal E_2_	0.001	0.007	0.009	1	0.926	1.001	0.988	1.014
Total number of retrieved follicles	0.043	0.030	2.002	1	0.157	1.044	0.984	1.107
Number of normally fertilized oocytes	-0.023	0.165	0.019	1	0.890	0.978	0.708	1.350
Number of fertilized oocytes with normal cleavage	-0.019	0.154	0.016	1	0.899	0.981	0.726	1.326
Constant	0.492	1.403	0.123	1	0.726	1.635		

Note: The presence or absence of syphilis, female age, BMI, duration of infertility, basal FSH, LH and E_2_ levels, total number of retrieved follicles, number of normally fertilized oocytes and number of fertilized oocytes with normal cleavage were considered as possible confounding factors and treated as continuous variables. The presence or absence of syphilis, female age and BMI were determined to be significant confounding factors.

B: partial regression coefficient

SE: standard error

df: degree of freedom

Exp(B): odds ratio

## Discussion

Age is related to ovarian response as well as the quality and quantity of embryos. Increased age appears to be a key predictor of worse assisted reproductive therapy (ART) outcome[28]. In our study, we matched the ages of the subjects in the control and syphilis groups to avoid the confounding influence of age on the results. Our results showed that on the day of hCG injection, the mean endometrial thickness of the syphilis group was significantly higher than that of the control group. Moreover, the clinical pregnancy rate of the syphilis group was significantly lower than that of the control group. These results imply that the endometrial thickness of the syphilis group on hCG injection day was not conducive to pregnancy. Studies have shown that a triple-line pattern combined with an optimal endometrial thickness (7–14 mm) is associated with a positive pregnancy outcome[29]. Okohue JE et al.[30] have reported that the pregnancy rate is significantly higher in women with an endometrial thickness of 7–14 mm compared with those with a thickness of >14 mm. They suggested that endometrial thickness affects the clinical pregnancy rate, potentially due to infection and inflammation caused by endometrial damage. Syphilis (as an STD)[31] can cause pelvic inflammatory diseases[32], which can lead to endometrial damage. Furthermore, this damage results in repeated repair and regeneration processes, which may thicken the endometrial lining and lead to abnormal endometrial functioning and reduced endometrial receptivity, resulting in infertility[33,34]. In addition, the numbers of normally fertilized oocytes and normally cleaved oocytes as well as the implantation rate were significantly lower for the syphilis group compared with the control group, and these findings may be related to pelvic inflammation, which may reduce the implantation rate[35]. Improvements in endometrial receptivity may increase the clinical pregnancy rate achieved by ART[36]. Therefore, physicians should assess the endometrium of infertile syphilis patients. Appropriate examinations and treatments for endometrial thickness or morphological abnormalities should be performed to improve clinical outcome.

The objective of IVF is to produce a healthy live born baby. In our study, one deformity was detected in the offspring of the subjects in the syphilis group, while none were observed in those of the subjects in the control group; however, due to the limited sample size, there were no significant differences between the two groups, further study is necessary to better characterize the deformity rate. Syphilis can cause miscarriage[37], but our results did not show significant differences in early miscarriage and the average fetal gestational period between the syphilis and control groups. These results may have been due to the systemic and standard anti-syphilis treatments that the syphilis patients had received prior to IVF treatment. Importantly, the number of mature oocytes and the rates of clinical pregnancy, biochemical pregnancy, and early miscarriage did not significantly differ among the subgroups, indicating that the IVF-related clinical outcomes of the couples with a positive history of syphilis were not influenced by whether the woman, man, or both were infected. Positive serology in the male group was associated with a lower offspring birth weight and shorter average gestational period compared with that in the female and couple groups; however, the reason for this finding is currently unclear.

Because most patients do not show clinical symptoms[38] and are not aware of their syphilis infection, the CDC recommends that individuals with multiple sexual partners should undergo a syphilis screening at least once per year[39]. All women should be tested for *Treponema pallidum* during their first prenatal examination and again late in pregnancy[40]. ART patients should receive routine syphilis serology testing. If the results are positive, the couples should receive standard anti-syphilis treatment[20], and be informed of the unfavorable IVF pregnancy outcome due to the infection. If the embryos require cryopreservation, a closed loading device should be used to avoid iatrogenic cross-infection in the hospital.

In addition, anti-cytokine antibodies (ACAs) and cytokines may also affect the fecundity of syphilis patients to a certain extent. ACAs are often found in the serum of these patients[41] and can inhibit the differentiation of cytotrophoblasts into syncytiotrophoblasts, thereby damaging cultivated blastocysts[42]. Cytokines are closely related to every aspect of the reproductive process and can be used to predict the clinical outcome of IVF[43]. Studies have shown that immunological changes occur in humans following *Treponema pallidum* infection; specifically, T helper cells are biased toward the Th1 phenotype in the peripheral blood of syphilis-infected individuals[44], and TNF-α secretion is significantly increased but is significantly reduced after treatment[45]. In a future study, changes in the ACA and cytokine levels should be assessed, which may represent an additional means of investigating the mechanisms underlying the influence of syphilis infection on ART success.

Limitations: Our syphilis group was composed of 160 patients, the sample sizes of each of the three syphilis subgroups were small, which may explain the lack of significant differences observed among them for most of the parameters tested.

## Conclusions

A positive history of syphilis infection can reduce the clinical pregnancy rate following IVF/ICSI. We have shown that this reduction in the clinical pregnancy rate involves a variety of factors, including endometrial thickness, the number of mature oocytes, the number of fertilized, normally cleaved oocytes, and the implantation rate. However, the mechanisms underlying the effects of syphilis on IVF success are currently unclear. Future studies should use larger sample sizes to better elucidate these mechanisms.

## Capsule

Syphilis infection reduces the clinical pregnancy rate after IVF/ICSI. The endometrial thickness and implantation rate of infertile couples treated for syphilis infection may be the most important factors affecting IVF outcome.

## Supporting Information

S1 File Strobe checklist of our manuscript. (DOC)

S2 File Translation of hospital ethics committee approval. (DOC)

S3 File The new editorial certificate. (DOC)
