# Supplementary material for: Clinical Outcomes of In Vitro Fertilization among Chinese Infertile Couples Treated for Syphilis Infection
Source: PLoS One. 2015 Jul 24;10(7):e0133726. doi: 10.1371/journal.pone.0133726 (PMC4514756; doi:10.1371/journal.pone.0133726)
Supplement: S1 File — (DOC) [file pone.0133726.s001.doc]

**1. S1. STROBE checklist of our manuscript:**

***1. Title***

Clinical Outcomes of IVF among Chinese Infertile Couples Treated for Syphilis Infection

**Type of study：**

A retrospective matched case-control study

***Abstract：***

Yes, it was showed in “Abstract” section. Please see manuscript.

***Introduction:***

1. ***Background/rationale***

Syphilis can cause reproductive tract infections that can induce inflammatory disease and result in infertility.The relationship between syphilis and infertility has been rarely reported in the literature.Therefore, we performed a retrospective analysis of the patients who received IVF/ICSI at Sun Yat-Sen Memorial Hospital to evaluate the effects of syphilis on IVF-ET outcomes.

***3. Objectives***

Evaluate the clinical pregnancy rate of infertile patients without syphilis who was compared with that of patients with syphilis after both groups received in vitro fertilization and embryo transfer（IVF-ET）and after the second group received the standard therapy for sexually transmitted diseases.

***Methods***

***4. Study design***

In this study, we retrospectively analysed the clinical data of 320 (syphilis infected group and control group) infertile patients who received IVF/ICSI treatment between January 2008 and March 2014 at the Reproductive Medical Center of Sun Yat-Sen Memorial Hospital.The couples with prior syphilis infection were also divided into three subgroups for comparison as follows: infected males, infected females, and infected couples.The clinical pregnancy rate was considered to be a measure of successful IVF outcome.
***5~6. Setting and Participant***

We used the patient histories recorded in the Sun Yat-sen Memorial Hospital medical record library for women who received IVF/ICSI treatment between January 2008 and March 2014 . The diagnosis of syphilis infection was confirmed by RPR and TPPA test.

***7. Variables***

Variables were showed in table1to table4.

***8. Data source/measurement***

Data collection: We performed A retrospective matched case-control analysis of the the case records from the medication doses during IVF treatment, number of oocytes retrieved, fertilisation rate, embryo development, pregnancy rate,early miscarriage rate, fetal birth weight,average gestational period and average newborn body lengths.

All the assessments were performed by experienced professional laboratory personnel who worked in the same hospital. The scores from their operation records were comparable.

***9. Bias***

The gonadotrophins dose was different during ovarian stimulation. The main reason is that we determined the starting dose based on the patient’s age and the number of antral follicles. We observed the development of follicles and ensured there were three follicles with ≥16 mm diameters, two follicles with ≥17 mm diameters, or one follicle with a ≥18 mm present in the ovaries bilaterally to prevent oocyte over- or under-development.

The syphilis patients enrolled in the study were infertility population, which were different from the general population.

***10. Study size***

A total of 320study cases were initially enrolled. 160 patients with syphilis infection comprised the syphilis group, and the control group consisted of patients without syphilis infections who were matched by age according to the ratio of 1:1

***11. Quantitative variables***

Patients age, basal FSH, basal LH, basal E2, E2 on HCG day,endometrial thickness on

HCG day,Gn dose, numbers of oocytes, normal rate of fertilization,normal rate of embryo cleavage, rate of usability embryo, fetal birth weight,average gestational period and average newborn body lengths.

**Non-normally distributed datasets**

Top quality embryos,numbers of embryos transferred

**Qualitative data**

implantation rate, pregnancy rate,early miscarriage rate, biochemical pregnancy rate.

***12. Statistical methods***

The collected data were analyzed using SPSS 13.0. The 3,275 couples were divided into 20 groups based on age (24-43 years). Random patient numbers were generated at each age level by SPSS 13.0. 160 cases were extracted using stratified random sampling from the randomly generated numbers in ascending order to form the age-matched group (control group). The t-test, the Mann-Whitney test, an analysis of variance, the least significant difference (LSD) test, the Kruskal-Wallis test, a chi-square analysis, multivariate analyses were conducted using. All results were considered significant at *P*<0.05.

***Results***

***13. Participants***

Data were collected from 320 couples in this study, including 122 suffering from primary infertility, and 198 suffering from secondary infertility. the data results of syphilis group (n=160) and control groups (n=160).We divided the 160 cases into three subgroups; women with syphilis infection(65 cases), men with syphilis infection(69 cases), and couples with syphilis infection(26 cases)

***14. Descriptive data***

See table1and table3 , the baseline and cycle characteristics of the patients, no missing values

***15. outcome of data***

Table 2 and table 3

***16. Main results***

pregnancy rate

***17. Other analysis***

No. of oocytes, fertilization rate, cleavage rate, dose of Gn.

***Discussion***

***18. Key results***

Syphilis infection reduced the clinical pregnancy rate after IVF/ICSI

***19. limitations***

Our syphilis group was composed of 160 patients. Although this number was above the minimum required sample size, each of the three syphilis subgroups had small sample sizes, which may explain the lack of significant differences observed among them with regard to most of the parameters tested.

***20. Interpretation***

A thickened endometrium decreases the clinical pregnancy rate achieved by ART, which may be due to the infection and inflammation caused by endometrial damage.Syphilis(as an STD)causes endometriosis which was not conducive to endometrial receptivity and reduced the clinical pregnancy rates.

***21. Generalisability***

ART patients should receive a routine syphilis serology test. If the results are positive, then the couples should receive standard anti-syphilis treatments.  physicians should assess the endometria of infertile patients with a history of syphilis infection who have received standard STD treatments, providing appropriate examinations and treatments for endometrial thickness or morphological abnormalities prior to performing ART, which may help to improve the IVF-related clinical outcomes in these patients.

***Other information***

***22. Funding***

This study was financially supported by the specialized research funds for the New Teachers Program at the Chinese Ministry of Education (20110171120083), the National Science Technology Research Projects of China (81100402), the Fundamental Research Funds for the Central Universities, and the Yat-Sen Scholarship for Young Scientists.

.

**References:** See manuscript
