# Supplementary material for: Clinical Outcomes of In Vitro Fertilization among Chinese Infertile Couples Treated for Syphilis Infection
Source: PLoS One. 2015 Jul 24;10(7):e0133726. doi: 10.1371/journal.pone.0133726 (PMC4514756; doi:10.1371/journal.pone.0133726)
Supplement: S2 File — (DOC) [file pone.0133726.s002.doc]

**S2 File.** **Translation of hospital ethics committee approval**

**Hospital ethics committee approval in Chinese:**

**
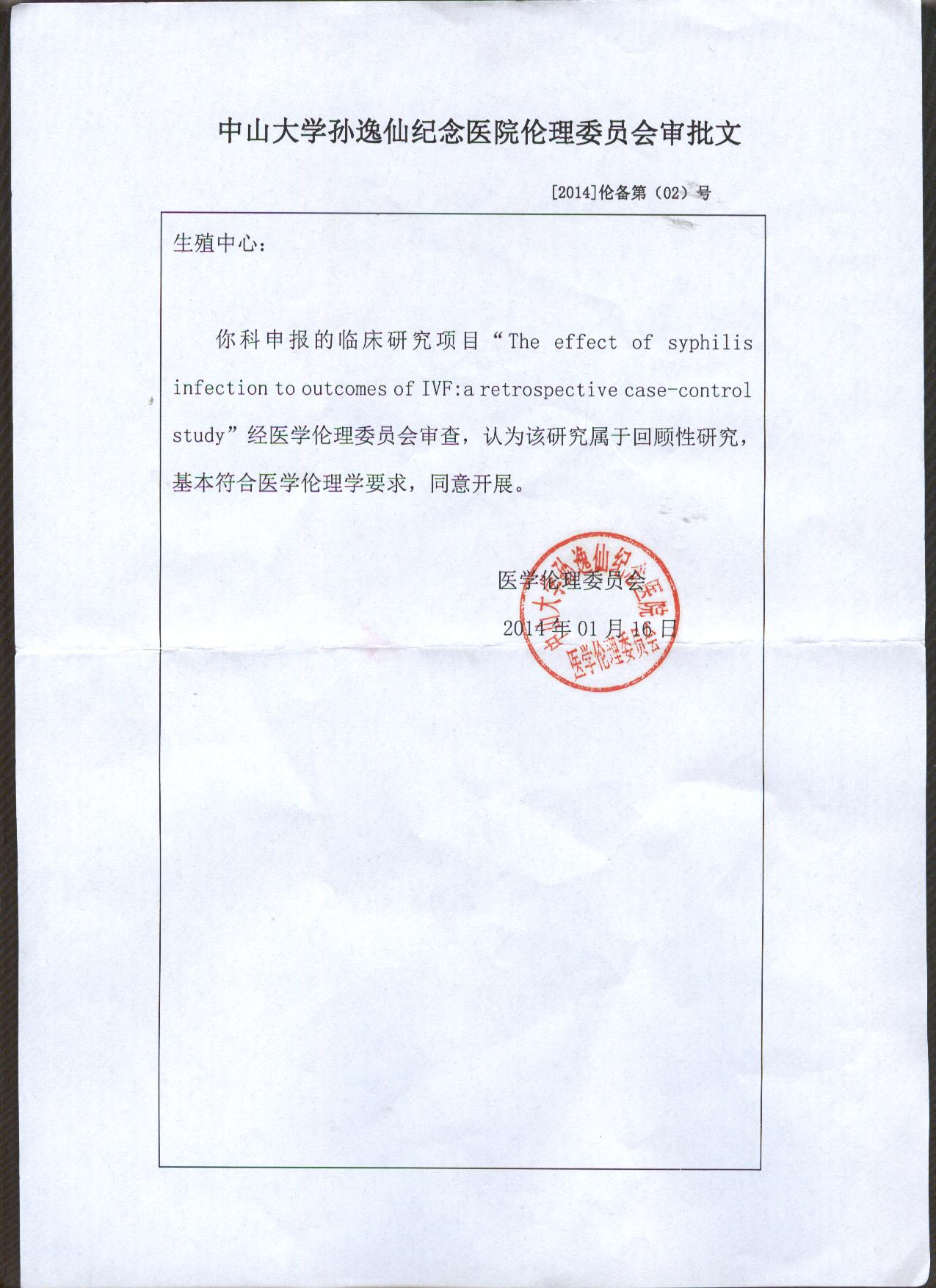
**

**Translation:**

**The ethics committee approval of the Sun Yat-sen Memorial Hospital**

[2014] Record of ethics committee No. (02)

Reproductive medicine centre：

After censored by the medical ethics committee of the Sun Yat-sen Memorial Hospital, the clinical research project of your department “The effect of syphilis infection to outcomes of IVF: a retrospective case-control study” was approved to be carried out based on the retrospective study according with the requirements of medical ethics.

Medical Ethics Committee

16 January 2014
