# Supplementary figures and images for: Clinical Outcomes of In Vitro Fertilization among Chinese Infertile Couples Treated for Syphilis Infection
Source: PLoS One. 2015 Jul 24;10(7):e0133726. doi: 10.1371/journal.pone.0133726 (PMC4514756; doi:10.1371/journal.pone.0133726)

**S3 File. The new editorial certificate**

**
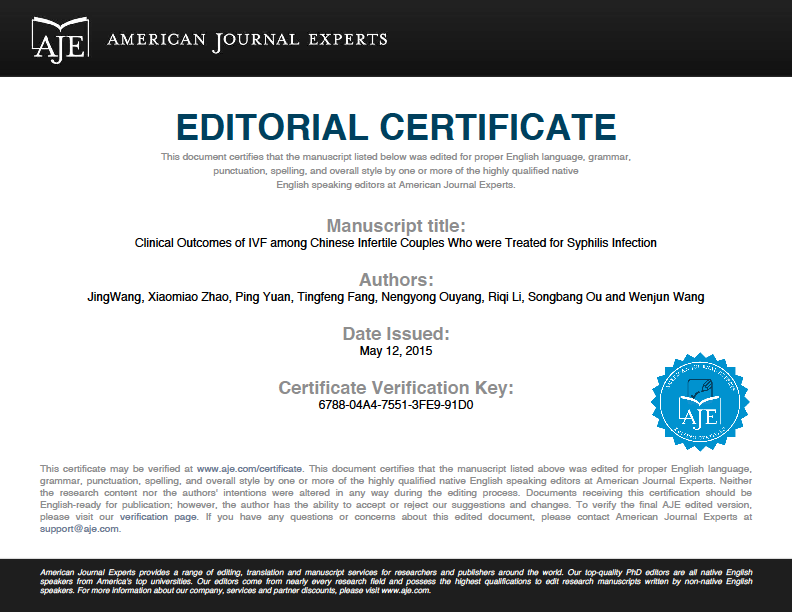
**

Supplement: S3 File — (DOC) [file pone.0133726.s003.doc]
